# Cosmetic “Gold Thread Therapy”: A Nuisance That Decreases the Diagnostic Quality of a Dental Panoramic Image

**DOI:** 10.1155/2021/4077930

**Published:** 2021-11-05

**Authors:** Mah Eng Ching, Lim Zhi Yin Joan, Phrabhakaran Nambiar

**Affiliations:** ^1^Department of Orthodontics, Faculty of Dentistry, MAHSA University, Bandar Saujana Putra, 42610 Selangor, Malaysia; ^2^Department of Restorative Dentistry, Faculty of Dentistry, MAHSA University, Bandar Saujana Putra, 42610 Selangor, Malaysia; ^3^Department of Oral Biology and Biomedical Sciences, Faculty of Dentistry, MAHSA University, Bandar Saujana Putra, 42610 Selangor, Malaysia; ^4^Department of Oral & Maxillofacial Clinical Sciences, Faculty of Dentistry, University of Malaya, 50603 Kuala Lumpur, Malaysia

## Abstract

During routine imaging of the craniofacial region, it is recognised that some “cosmetic” procedures with metallic insertions can be revealed radiographically. These objects however make it difficult to obtain a good interpretation of anatomical structures for management of diseases. A 58-year-old female patient visited a private dental facility in Kuala Lumpur for prosthodontic replacement of missing teeth. The dental panoramic image revealed generalized bone loss and numerous unusual multiple thread-like radioopacities. These gold threads made radiographic evaluation difficult and complicated the process of treatment planning for dental implant placement advocated for this patient.

## 1. Introduction

Digital intraoral images along with other radiographic projection methods such as dental panoramic images, extraoral imaging views, and cone-beam computed tomography (CBCT) scans are employed to diagnose diseases or abnormalities in the craniofacial region. Therefore, any fault or artifact observed on these images affects our interpretations and ultimately the diagnosis. Different types of radiopaque images and artifacts caused by accessory piercings, tongue studs, necklaces, reading glasses, removable prostheses, and earrings have been described. To avoid having these faults on the images, it is essential to remove all metallic objects in the maxillofacial region. However, there are metallic dental materials that cannot be removed. These include amalgam fillings, implants, and metallic braces that cause acquired artifacts on the images [1].

During routine imaging of the craniofacial region, it is recognised that some “cosmetic” procedures can also be demonstrated radiographically. Unexpected foreign metallic talismans inserted into the body, often referred to as “susuk” (charm needles) or acupuncture needles hidden in the body, might become revealed [2–4]. These objects make it difficult to obtain a good interpretation of images for management of the cases. They will also cause scattering on CBCT images and affect the treatment planning for implant placements [5]. In many occasions, the nonexperienced dental practitioner can mistake these faults for electrostatic discharges (on X-ray films), ghost images, or even radiopaque images of orthodontic ligature wires [6].

Another less reported hyperdense, short, thread-like fragments noted on oral and maxillofacial images/radiographs are caused by gold thread therapy or lift. This is also one of the methods of facial rejuvenation to firm up sagging skin in the cheek and jaw line by threading a web of gold fragments in the subdermal soft tissues [7]. As this procedure of face liftings is getting popular, the dental practitioners should be aware of the radiographic appearance of these foreign bodies to avoid any misinterpretation for diagnosis. Patient management will be further improved, and embarrassment will be avoided if these patients are approached gently with tact as the practitioner is aware of this therapy.

## 2. Case Report

A 58-year-old female patient visited a private dental facility in Kuala Lumpur for replacement of missing teeth and other prosthodontic treatment. Medical history of the patient revealed that the patient was unremarkable. Patient had her teeth extracted because of caries. An extraoral examination of the head and neck region showed no obvious abnormalities. Intraoral examination revealed a few missing teeth and retained root in the maxilla, and quite a few prosthodontic procedures were done in the mandible. Patient was interested to have dental implants done to replace the missing teeth. A dental panoramic image was taken for radiographic investigation. The Pax-i Digital Panoramic Unit (Vatech Imaging Systems, Korea) with the following parameters were used: tube voltage: 74 kVp, tube current: 10 mA, and scan time: 13 secs. The image revealed generalized bone loss and innumerable unusual multiple thread-like radioopacities over a wide area. These fragments were observed located bilaterally over the lower border of the orbits and middle and lower facial soft tissues. Radioopacities were very thin and tortuous and have short fragments.

Patient was enquired about whether she had undergone any kind of surgery before on her face. The patient revealed she had undergone “gold thread therapy” for facial “rejuvenation” (on multiple occasions) during the last 10 years. She mentioned that during the face-lift procedure, very thin needle with the gold thread was inserted under her skin, forming a mesh beneath it. The entire procedure was done under local anaesthesia and took 30 minutes to 1 hour. She also mentioned that she was very satisfied with the results of the procedure. These gold threads were inserted by a foreign medical doctor. The doctor carried his own instruments and visited households where patrons needed this service. All treatment is considered private and confidential and was also illegal as the doctor is not registered with the Malaysian Medical Council. The patient ordered for the service through a cosmetic agent, and procedures were performed whenever the doctor was visiting Malaysia.

The patient is a grandma with children and grandchildren; however, she still looks like a youthful middle-aged woman with firm facial muscles. She appeared to be confident and had high self-esteem. She admitted she has got addicted with the procedure and is very satisfied with it. She did not seem mindful if the doctor is not registered with the national medical council nor his procedure is considered an illegal practice.

## 3. Discussion

Gold is preferred metal of choice for this therapy as it is biocompatible and is not rejected by human skin and underlying tissues. It can be safely left in place for a long-term face lifting and subsequent skin rejuvenation. The “golden thread lift” was first introduced in dermatology by Dr. Caux in France approximately 30 years ago and then developed in Russia by Dr. Pawel Koziczynski in year 2000 [7–9]. Since then, it has also been adopted and broadly advertised in western countries and Asia. The early procedures involved the implantation of short 0.5 mm diameter gold thread. These procedures were very invasive, had a longer, less appealing recovery time, and required local anaesthesia most of the time.

Nowadays, microfine surgical gold threads (0.1 mm diameter, 24 K gold) are implanted into the subdermal tissues. With the finer/smaller diameter of gold thread, no local anaesthesia is needed [7, 8, 10]. A gold web is weaved to hold the facial tissues in place, provide mechanical support to the tissues, and prevent sagging of the skin. The gold will rejuvenate the skin by initial reaction within the skin tissues to coat the threads with collagen and intensify angiogenesis and blood circulation of the surrounding tissue. The usual sites for gold thread implantation in the face are cheeks, forehead, and mouth. The threads can also be inserted in other parts of the body such as hand, arms, and hips [7, 11]. In addition, in this case, the gold thread lift was not only performed in the cheek and perioral area but also at the periorbital area.

**Figure 1:**
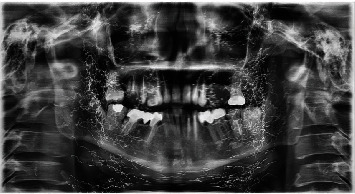
Numerous, short, tortuous, hyperdense threads revealed bilaterally in the maxillofacial region.

Few case reports about gold lift thread on images/radiographs have been reported in the literature, but the threads showed on the dental panoramic radiograph and periapical radiograph were fewer compared to what has noted in this case. In 2014, Mizrahi and Scully reported one of the earliest unusual linear radioopacities that were evident over a wide area on pantomographs [12]. Garg et al. described them as radiodense string-like artifacts in the posterior aspect of the maxilla and the mandible of a patient who confirmed undergoing gold thread facelift [9]. In addition, Cho and Park reported noticing gold thread therapy in posterior-anterior extraoral radiograph. According to them, this therapy was an adjunct for acupuncture for relieving perennial headaches in a 39-year-old Korean lady [13]. Recently, Negayama and Fujikawa provided a 3-dimensional volumetric rendering of a computed tomography (CT) scan of a patient who had undergone insertion of “gold threads” for facial rejuvenation. Their description of the insertions was very apt—numerous short, hyperdense, thread-like fragments, whose actual thickness was intensified, randomly distributed over the face [5]. Evidently bilateral symmetry in the gold lift was maintained so that the patient is aesthetically outstanding as the person in Figure 1.

Similar to gold thread lifting, implanting susuk subcutaneously is commonly done at the maxillofacial region for aesthetic purposes. Insertion of “susuk” or charm needles is a common practice in Malaysia. When a radiograph is taken for diagnostic purposes, these invisible implanted needles are typically observed by chance. These are often misdiagnosed as foreign bodies, acupuncture needles, fractured endodontic files, or even root fillings and restorative pins [2, 3]. Clinicians should be able to differentiate between these two foreign entities on the face. Gold threads can make radiographic evaluation more difficult because they can obstruct essential anatomical structures and complicate the process of treatment planning in dental procedure—in this case, it was for dental implant placement. A CBCT scan on this person will have limited benefit as there shall be numerous streaking, beam hardening, and cupping artifacts which will render the scan useless [1]. The efficiency of gold thread lifting used for plastic surgery or acupuncture is controversial [14]. The drawback of the gold thread face lift is that the gold threads can migrate and may get fragmented over a period of time.

In addition, a variety of absorbable (collagen, hyaluronic acid, calcium hydroxyapatite, and poly-L-lactic acid) and nonabsorbable (polymethylmethacrylate beads) materials have been developed as dermal fillers for the facial area for cosmetic purposes [15]. These mineralized particles shall be observed to have diverse radiodensities and varying shapes and therefore different from these gold threads.

## 4. Conclusion

The above case highlights the importance of clinical practitioners working in Southeast Asian countries being aware of such gold thread therapy to reduce adverse and unintended effects during diagnosis and treatment planning. Nowadays, cosmetic procedures in the facial region are getting common as aesthetic awareness increased for the past few decades. The general dental practitioner should be aware of facial rejuvenation procedures and their radiographic appearance. A comprehensive medical history taking of the patient should include questions about utilization of such therapies.
